# Exploration of the molecular mechanism of modified Danggui Liuhuang Decoction in treating central precocious puberty and its effects on hypothalamic-pituitary-gonadal axis hormones

**DOI:** 10.1186/s41065-025-00420-9

**Published:** 2025-04-08

**Authors:** Xiaqing Liu, Pinggan Li, Xiangna Yang, Ting Xie, Hua Xu

**Affiliations:** 1 Guangzhou University of Chinese Medicine, Guangzhou, 510405 Guangdong P. R. China; 2 Department of Traditional Chinese Medicine, Guangzhou Women and Children’s Medical Center, Guangzhou, 510620 Guangdong P. R. China; 3 Department of Children’s Endocrinology, Guangzhou Women and Children’s Medical Center, Guangzhou, 510620 Guangdong P. R. China; 4 Pediatrics of Traditional Chinese Medicine, The First Affiliated Hospital of Guangzhou University of Chinese Medicine, No. 16, Airport Road, Guangzhou, 510405 Guangdong P. R. China

**Keywords:** Central precocious puberty, Gonadotropin-releasing hormone, MDGLHD, Network Pharmacology, Molecular Docking

## Abstract

**Aim:**

To evaluate the molecular mechanism of modified Danggui Liuhuang Decoction (MDGLHD) in treating central precocious puberty (CPP).

**Methods:**

CPP-related genes were obtained from GEO dataset, MalaCard, DisGeNET and GeneCards databases. MDGLHT ingredients and targets were obtained in TCMSP, HERB, and SwissTargetPrediction databases. Protein-protein interaction (PPI) network was constructed and analyzed using STRING database and Cytoscape 3.9.1. Genetic ontological (GO) analysis and Kyoto Encyclopedia of Genes and Genomes (KEGG) pathway enrichment analysis were performed with DAVID and Metascape databases. Molecular docking was performed with PyMoL and AutoDock-Vina software. The GnRH secretion model was established by E2 induction of GT1-7 cells. CCK-8, ELISA and qRT-PCR were used to detect the effects of MDGLHD on gonadotropin-releasing hormone (GnRH) secretion and endocrine signaling receptor gene expression.

**Results:**

318 potential targets of MDGLHD in CPP treatment were screened out. Quercetin, kaempferol, and (S)-Canadine were considered to be the most important active ingredients in MDGLHD. Bioinformatics analysis showed that these targets were associated with response to hormone, JAK-STAT signaling pathway and HIF-1 signaling pathway. Quercetin, kaempferol, and (s)-Canadine had good binding affinity with tumor protein p53 (TP53), estrogen receptor 1(ESR1), Jun proto-oncogene (JUN), MYC proto-oncogene (MYC) and AKT serine/threonine kinase 1 (AKT1). In vitro experiments showed that MDGLHD extract can inhibit GnRH secretion and the expression of neuroendocrine signaling receptor protein gene.

**Conclusion:**

MDGLHD treatment of CPP is achieved through multi-components, multi-targets and multi-pathways, and inhibition of GnRH secretion and neuroendocrine signaling.

**Graphical Abstract:**

**Figure:**
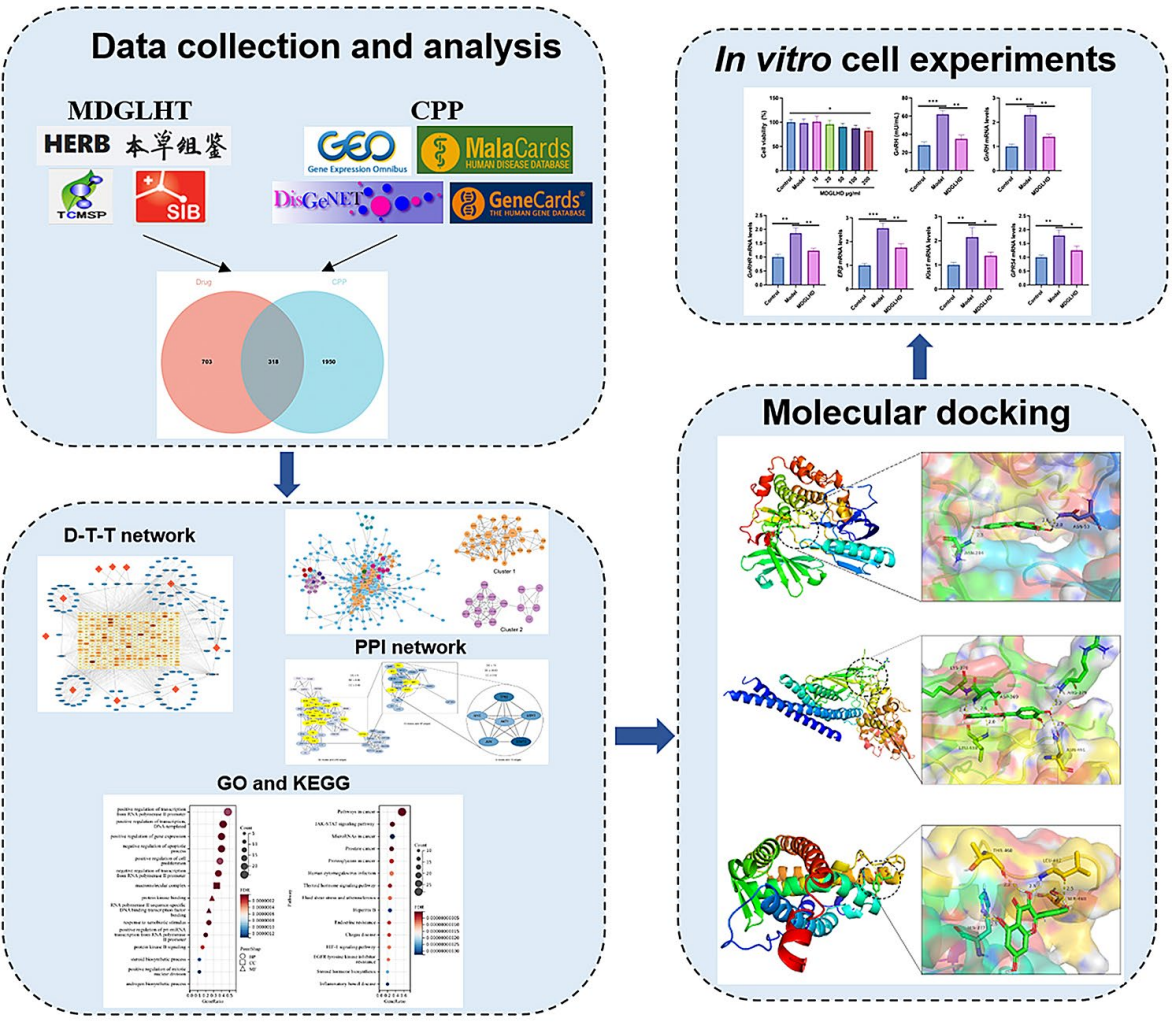

**Supplementary Information:**

The online version contains supplementary material available at 10.1186/s41065-025-00420-9.

## Introduction

Precocious puberty (PP) mainly manifests in the early appearance of secondary sexual characteristics [1, 2]. The total incidence of PP is estimated to be between 1:500 and 1:10000, and is predominantly female [3, 4]. Clinically, PP can lead to the early development of secondary sexual characteristics, such as progressive breast development in girls and increased testicular volume in boys, rapid bone maturation, growth rate, and premature epiphysis healing, which affects the final adult height of children [5, 6]. In recent years, epidemiological statistics have found that the morbidity of PP shows an increasing trend [3, 4, 7].

According to whether the hypothalamic-pituitary-gonad (HPG) axis is activated, PP is mainly divided into central precocious puberty (CPP) and peripheral precocious puberty (PPP) [8]. CPP resulting from premature of pulsating hypothalamic gonadotropin-releasing hormone secretion is considered to be the “true” PP. Gonadotropin-releasing hormone (GnRH) analogue (GnRHa) is a synthetic peptide drug, including buserelin, histrelin, leuprorelin, triptorelin and goserelin, which is often used to treat CPP [9, 10]. It binds to the GnRH receptor of anterior pituitary, and can briefly promote luteinizing hormone (LH) and follicle-stimulating hormone (FSH) in the early stage of administration, then suppress corresponding receptors in pituitary target cells, inhibit the pituitary-gonadal axis, and reduce the secretion of LH, FSH and gonadal hormones, thereby controlling the process of sexual development [11, 12].

Compared with the current treatment strategies, Traditional Chinese medicine (TCM) has certain advantages, including but not limited to high efficiency, safety and low toxicity. Many of the bioactive components in TCM show a wide range of biological activities including anti-inflammatory, antioxidant, immunomodulatory, anti-diabetic, neuroprotective and anti-cancer effects, and some prescriptions show promising effects in alleviating adverse reactions induced by current treatment strategies [13–17]. TCM has shown the potential to treat PP [18, 19]. A clinical study showed that ZiYin Xiehuo granules and Zishen Qinggan granules can both affect the size of breast, uterus, ovary and ovarian follicle in girls with PP [20]. Pomegranate extract can supplement and improve the efficacy of GnRHa in the treatment of idiopathic CPP in Chinese girls [21, 22]. A rat experiment demonstrated that both Ziyin Xiehuo Recipe and Yishen Tianjing Recipe could regulate the expression and secretion of hypothalamic GnRH, somatostatin (SS), growth hormone (GH), FSH and LH, et al. [23]. Previous studies have shown that Danggui Liuhuang Decoction (DGLHD) has pharmacologic effects such as anti-inflammatory properties, immunosuppression, insulin sensitization, hormone regulation, hypoglycemia and blood lipid reduction [24–27]. Importantly, several clinical trials have validated the safety of DGLHD in clinical application [28, 29]. However, the function and potential mechanism of Modified Danggui Liuhuang Decoction (MDGLHD) in CPP have not been reported. So, this study aims to explore the molecular mechanism of MDGLHD in the treatment of CPP by combination with bioinformatics, network pharmacology, molecular docking and in vitro experiments.

## Materials and methods

### Collection of CPP-related genes

Makorin ring finger protein 3 (MKRN3) has been identified as an inhibitor of puberty initiation, and its mutation loss is related to CPP [30, 31]. From Gene Expression Omnibus (GEO) (https://www.ncbi.nlm.nih.gov/geo/) GSE208722 dataset was downloaded. The dataset contained three MKRN3 wild-type human induced pluripotent stem cells (hiPSCs) and MKRN3-deficient hiPSCs cell lines. GEO2R online analytical tool (https://www.ncbi.nlm.nih.gov/geo/geo2r/) was used to analyze the differentially expressed genes (DEGs). Differential expression analysis was performed using DESeq2 package in R. *P* < 0.05 and Log_2_ fold change (logFC) values ≥ 1 were used to screen up-regulated genes; *P* < 0.05 and logFC values≤-1 was applied to screen down-regulated genes. With the “Central precocious puberty” as the keyword, databases including MalaCard (https://www.malacards.org/, relevance score ≥ 0.7), DisGeNET (http://www.disgenet.org/, v.24.3, C0342543 and C0342544, score ≥ 0.01) and GeneCards (https://www.genecards.org/, v.5.19, relevance score ≥ 0.7) were searched to retrieve CPP-related genes. All genes encoding proteins were included.

### Acquisition of the ingredients of MDGLHD and their targets

Traditional Chinese Medicine Systems Pharmacology Database and Analysis Platform (TCMSP, http://tcmspw.com/tcmsp.php) and HERB database (http://herb.ac.cn/) were used to get the potential functional bioactive components of MDGLHD, with oral bioavailability (OB) ≥ 30% and drug similarity (DL) ≥ 0.18 as the screening conditions [32]. Through the PubChem database (https://pubchem.ncbi.nlm.nih.gov/), SMILES files of the components were obtained, and the targets of the components were predicted by SwissTargetPrediction database (http://www.swisstargetprediction.ch/).

### Network construction

Drug targets and CPP-related genes were further screened with a Venn diagram to obtain MDGLHD’s targets in CPP treatment. Cytoscape 3.8.0 software was used for the construction of drug-compound-target network. The targets of MDGLHD were imported into STRING database (https://string-db.org/), and the protein-protein interaction (PPI) network was obtained. The species was set to “Home Sapiens”, and the minimum interaction threshold was set to “confidence > 0.9”, and the rest were set to “default values”. After the PPI network was constructed, the MCODE and CytoNCA plugins were used to filter important PPI network modules and calculate degree centrality (DC), betweenness centrality (BC) and closeness centrality (CC).

### Gene oncology (GO) and Kyoto encyclopedia of genes and genomes (KEGG) enrichment analyses

The Database for Annotation, Visualization and Integrated Discovery (DAVID) (https://david.ncifcrf.gov/summary.jsp) [33] and Metascape database (http://metascape.org/gp/index.html) [34] were applied for GO analysis (cellular components, molecular function and biological processes) and KEGG enrichment analysis.

### Molecular Docking

mol2 files of the 3D structure of the active ingredients of MDGLHD were downloaded from the RCSB PDB database (https://www.rcsb.org/). The components were hydrotreated with AutoDocktools software (v.1.5.7), and the protein was hydrotreated, and water molecules were removed, and exported as pdbqt file. The pdbqt structures of receptors and ligands were introduced to AutoDock software. When constructing a mating box, spacing (angstrom) was set to 1, center was set on the macromolecule, and numbers of points in x-, y-, and z-dimension were set to keep the protein completely wrapped by box. For each protein, these parameters of the docking box were defined by a configuration (config) file for further docking by AutoDock Vina, which included the center of the box, the size of the box (config.txt), the maximum number of binding modes to output (num modes) (set to 10), energy_range (set to 3) and exhaustiveness (set to 8). The molecular docking was performed by using AutoDock Vina software (v.1.5.7), and the docking affinity was calculated. The contributions of various forces were considered when calculating the binding energy, including electrostatic interactions between molecules, van der Waals forces, hydrogen bonds, hydrophobic interactions, etc. The result with the lowest binding energy was selected for visualization through PyMoL software (v.2.4.0).

### Cell culture

Mouse hypothalamic neuron GT1-7 (BFN60804021) was purchased from BLUEFBIO Life Sciences (Shanghai, China). The cells were cultured in Dulbecco modified Eagle medium (DMEM; Sigma-Aldrich, Shanghai, China) containing 10% fetal bovine serum (FBS; Gibco, Carlsbad, CA, USA) and 1% penicillin-streptomycin (Gibco, Carlsbad, CA, USA). The cells were placed in an incubator containing 5% CO_2_ at 37 °C. First, the cells were divided into two groups, including control group and model group. According to previous studies, to construct an in vitro model of CPP, the cells in the model group were cultured overnight in DMEM containing 100 pmol/L estradiol (E2) [18]. The control group was treated with the same volume of dimethyl sulfoxide (DMSO, Sigma-Aldrich, St. Louis, MO, USA). In the toxicity test, the model group was divided into 6 groups, and the cells were treated with E2 for 24 h, and then treated with 0, 10, 20, 50, 100, and 200 µg/ml MDGLHD extract for 24 h respectively. For the GnRH and gene expression test, three groups were set: control group, model group and MDGLHD treatment group. For MDGLHD treatment group, the cells were treated with E2 for 24 h, and then treated with 100 µg/ml MDGLHD extract for 24 h.

### Preparation of MDGLHD extract

Angelica 6 g, raw rehmannia 6 g, cooked rehmannia 6 g, astragalus 12 g, coptis 6 g, radix scutellariae 6 g, cortex phellodendri 6 g, rhizoma anemarrhenae 6 g, cortex moutan 6 g and grilled turtle plate 6 g were obtained from The First Affiliated Hospital of Guangzhou University of Chinese Medicine. The medicinal materials mentioned were mixed, immersed in 2 L water, and decocted twice for 1.5 h each time. After decoction, the supernatant was collected after centrifugation. The supernatants obtained were then mixed and evaporated to obtain MDGLHD powder. The MDGLHD powder was then dissolved in dimethyl sulfoxide (DMSO; Sigma-Aldrich, St. Louis, MO, USA), and diluted by the medium for the subsequent experiments.

### Cytotoxicity test

Cell counting kit-8 (CCK-8, Solarbio, Beijing, China) was used to detect the toxicity of MDGLHD extract on GT1-7 cells. GT1-7 cells (1 × 10^3^ cells/well) were inoculated into a 96-well plate. The cells were incubated with 10, 20, 50, 100, and 200 µg/ml MDGLHD extract for 24 h, and then treated with 10 µL CCK-8 solution in each well for 2 h. Finally, the absorbance of each well at 450 nm wavelength was detected by a microplate reader.

### Enzyme-linked immunosorbent assay (ELISA)

A mouse GnRH ELISA kit (CB10243-Mu, COIBO BIO, Shanghai, China) was used to detect GnRH levels. The supernatant of cell culture medium was collected, and then the experiment was carried out according to the manufacturer’s instructions. The absorbance of each well at 450 nm was detected with a microplate reader. The concentration of each sample was calcuated according to the standard curve.

### RNA extraction and quantitative real-time polymerase chain reaction

GT1-7 cells treated with E2 and MDGLHD extract were collected and total RNA was extracted from the cells using TRIzol reagent (Thermo Fisher Scientific, Shanghai, China) according to the manufacturer’s protocol. RNA was converted to cDNA using the FastKing cDNA first strand synthesis kit (KR116, TIANGEN, Beijing, China). qRT-PCR was performed using the FastKing one-step RT-PCR kit (KR123, Tiangen, Beijing, China) on ABI 7900 rapid real-time fluorescent quantitative PCR system (ABI, CA, USA). As an endogenous gene, GAPDH was analyzed using the 2^−ΔΔCT^ method [35]. Primers used in this study are listed in Table 1.

**Table 1:** Primer sequences used in quantitative real-time polymerase chain reaction

Gene	Forward primer (5’-3’)	Reverse primer (3’-5’)
*GnRH*	AGCACTGGTCCTATGGGTTG	GGGGTTCTGCCATTTGATCCA
*GnRHR*	TGC​AGG​ACC​ACA​GAA​CTA​CAG	GTC​CAG​CAG​ACG​ACA​AAG​GA
*ERβ*	CCCTGCTGTG ATGAATTACAG	TCGGTTCCCACTAACCTTCC
*Kiss1*	GAT​GTC​TGC​AGC​CTG​AGT​CCC	AGG​CAT​TAA​CGA​GTT​CCT​GGG
*GPR54*	TACATCCAGCAGGTCTCGGTG	ACGTACCAGCGGTCCACACT
*GAPDH*	AGGTCGGTGTGTGAACGGATTTG	GGGGTCGTTGATGGCAACA

### Statistical analysis

All of the in vitro assays were performed in triplicate, and repeated for at least three times. All data analyses were performed using GraphPad Prism 7 (GraphPad software, Inc. CA, USA). All experiments were independently conducted three times and the data were expressed as “mean ± standard deviation (SD)”. The data among multiple groups were compared by one-way analysis of variance (ANOVA), followed by Tukey’s post hoc test. *P* < 0.05 was statistically significant.

## Results

### Screening of CPP-related genes

**Fig. 1:**
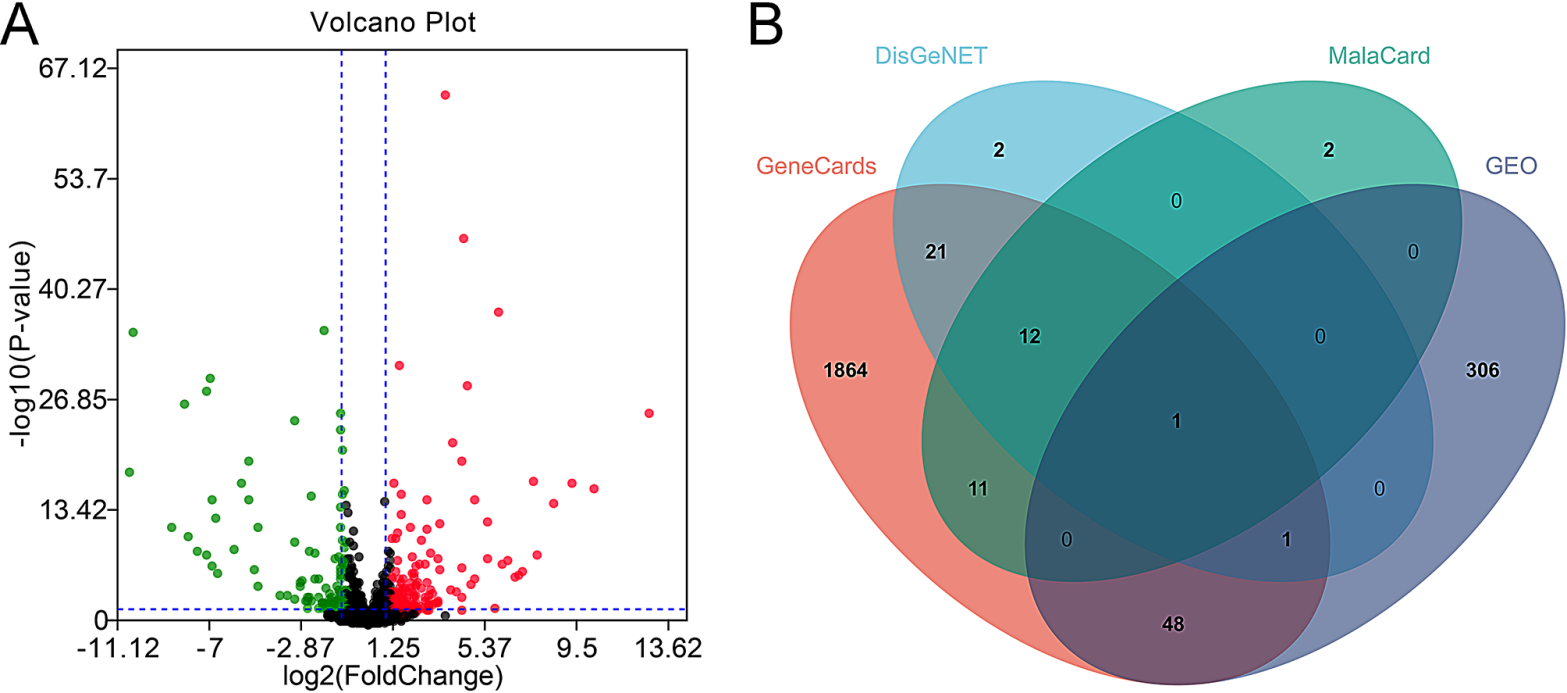
Screening of CPP-related genes. **A**. The volcano map shows DEGs in GSE208722 dataset. Green represents down-regulated genes and red represents up-regulated genes. **B**. The Venn diagram shows the CPP-related genes are identified by MalaCard, DisGeNET, GeneCards databases and analysis of GSE208722

First of all, we analyzed the DEGs in the GSE208722 dataset. 356 DEGs were obtained, of which 207 genes were up-regulated and 149 genes were down-regulated (Fig. 1A). 26, 37 and 1958 genes were obtained from MalaCard, DisGeNET and GeneCards databases, respectively, and a total of 2268 CPP-related genes were obtained after integration and removal of the duplicates (Fig. 1B).

### Components and targets of MDGLHD

**Fig. 2:**
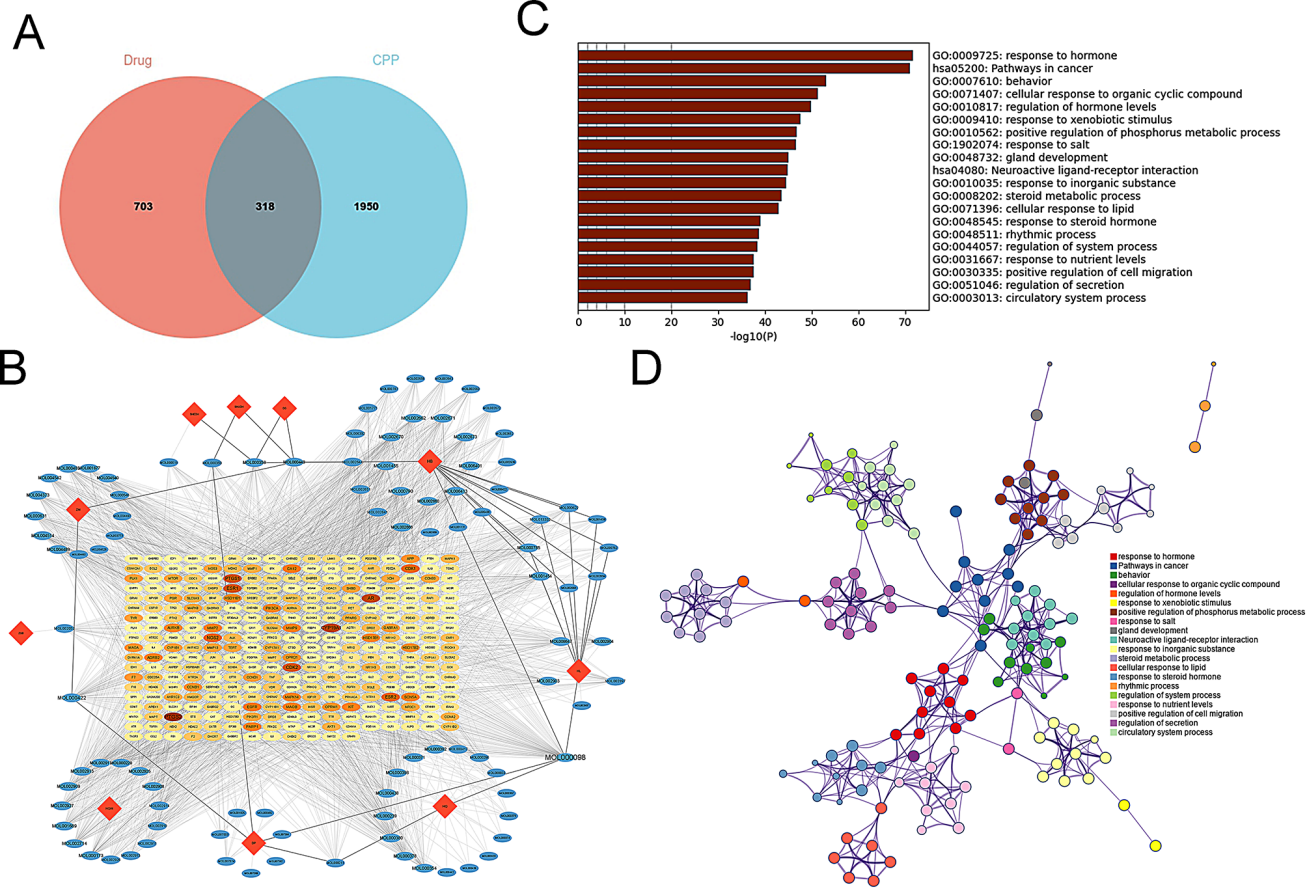
Components and targets of MDGLHD. **A**. The Venn diagram is applied to obtain the target genes of MDGLHD in CPP treatment. **B**. Cytoscape 3.8.0 software was used for the construction of drug-compound-target network. The yellow and orange oval nodes represent the targets. The blue oval nodes represent pharmaceutical ingredients. The orange diamond-shaped nodes represent medical materials. HQ, Huangqi (astragalus); HB, Huangbai (cortex phellodendri); HL, Huanglian (coptis); HQIN, Huangqin (radix scutellariae); DG, Danggui (angelica); ZM, Zhimu (rhizoma anemarrhenae); DP, Danpi (cortex moutan); ZGB, Zhiguiban (grilled turtle plate); SHUDH, Shudihuang (cooked rehmannia); SHEDH, Shengdihuang (raw rehmannia). **C**-**D**. Histogram and interactive network show the results of GO analysis and KEGG enrichment analysis. The same color of the nodes in the network represents a term, and the node size represents the number of genes in the term

According to OB ≥ 30% and DL ≥ 0.18, 99 active ingredients were screened from the medincal materials of MDGLHD (Table S1). Then potential targets of 99 components were searched through TCMSP and SwissTargetPrediction database. Five of the components (kihadanin A, Chrysanthemaxanthin, 4-O-methylpaeoniflorin_qt, mudanpioside-h_qt and paeonidanin_qt) did not predict any targets. The remaining 94 components had a total of 1021 targets. Combined with CPP-related genes, 318 potential targets for MDGLHD in CPP treatment were identified (Fig. 2A). The ingredients and potential targets for MDGLHD in CPP treatment were imported into Cytoscape 3.7.2 software to obtain the network, which included 427 nodes and 2939 edges (Fig. 2B). Among these bioactive ingredients, quercetin (MOL000098, DC = 122, BC = 33528.03, CC = 0.44), kaempferol (MOL000422, DC = 70, BC = 5621.57, CC = 0.40), and (S)-Canadine (MOL001455, DC = 51, BC = 4501.71, CC = 0.39) were considered to be the most important bioactive ingredient. A subsequent analysis with Metascape database showed that these 318 targets were related with “response to hormone”, “pathways in cancer”, “behavior”, “cellular response to organic cyclic compound” and “regulation of hormone levels” (Fig. 2C-D), which strongly suggested that MDGLHD may treat CPP by regulating hormone secretion.

### PPI network construction and core target screening

**Fig. 3:**
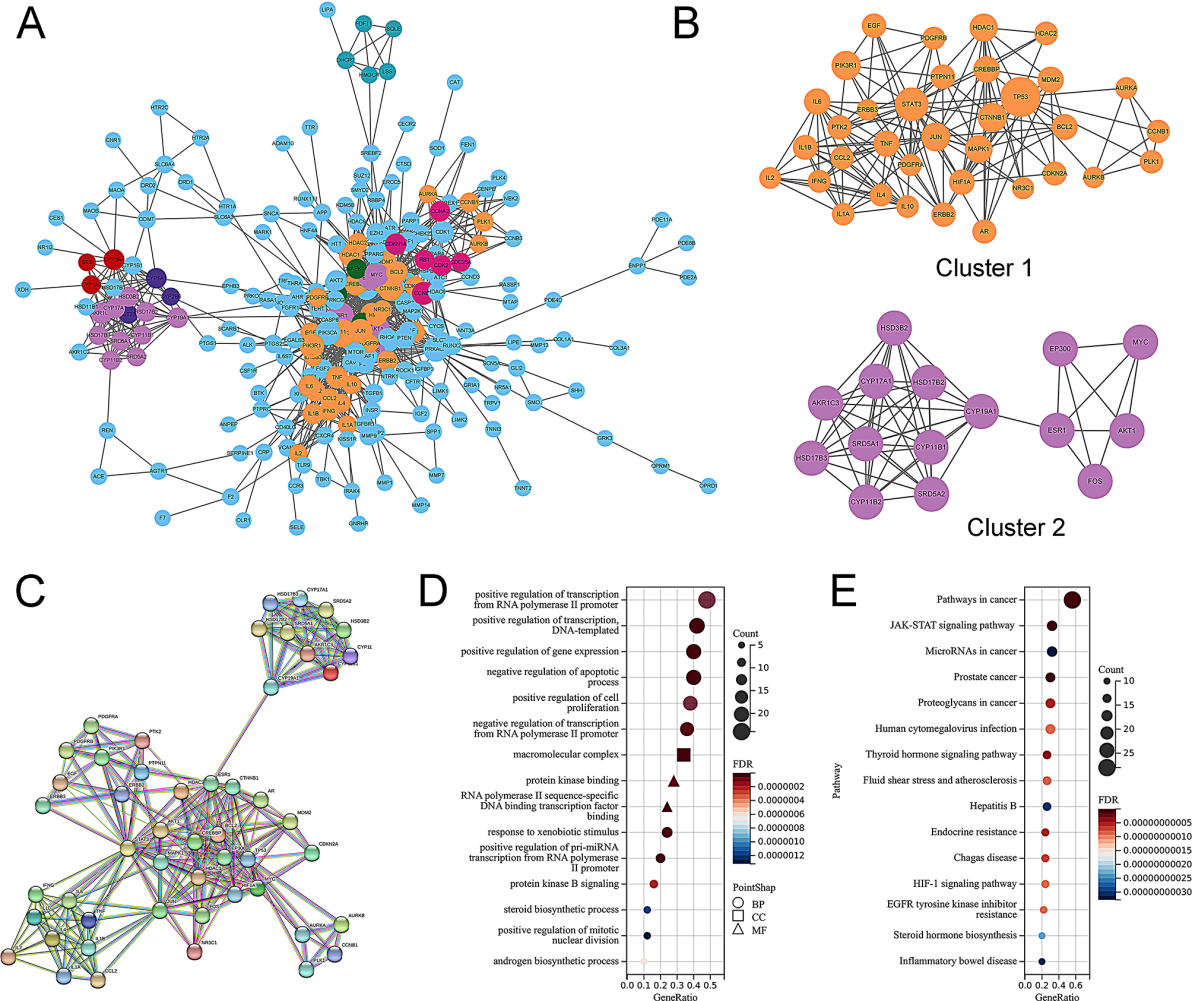
PPI network construction and key module screening. **A**. Cytoscope software was used to visualize the PPI network of targets. Different colors represent different clustering modules, orange represents cluster 1 (score: 8.353, including 35 nodes and 142 edges); Light purple represents cluster 2 (score: 7.857, including 15 nodes and 55 edges); Cyan represents cluster 3 (score: 4.500, including 5 nodes and 9 edges); Pink represents cluster 4 (score: 4.400, including 6 nodes and 11 edges); Red, green, and purple represent clusters 5, 6, and 7 respectively (score: 3.000, including 3 nodes and 3 edges). **B**. Cluster 1 and cluster 2 subnetworks. **C**. STRING database was applied to construct the PPI network of the 50 genes obtained from cluster 1 and cluster 2. **D** & **E**. GO analysis and KEGG enrichment analysis were applied to predict the biological functions of the 50 genes

**Fig. 4:**
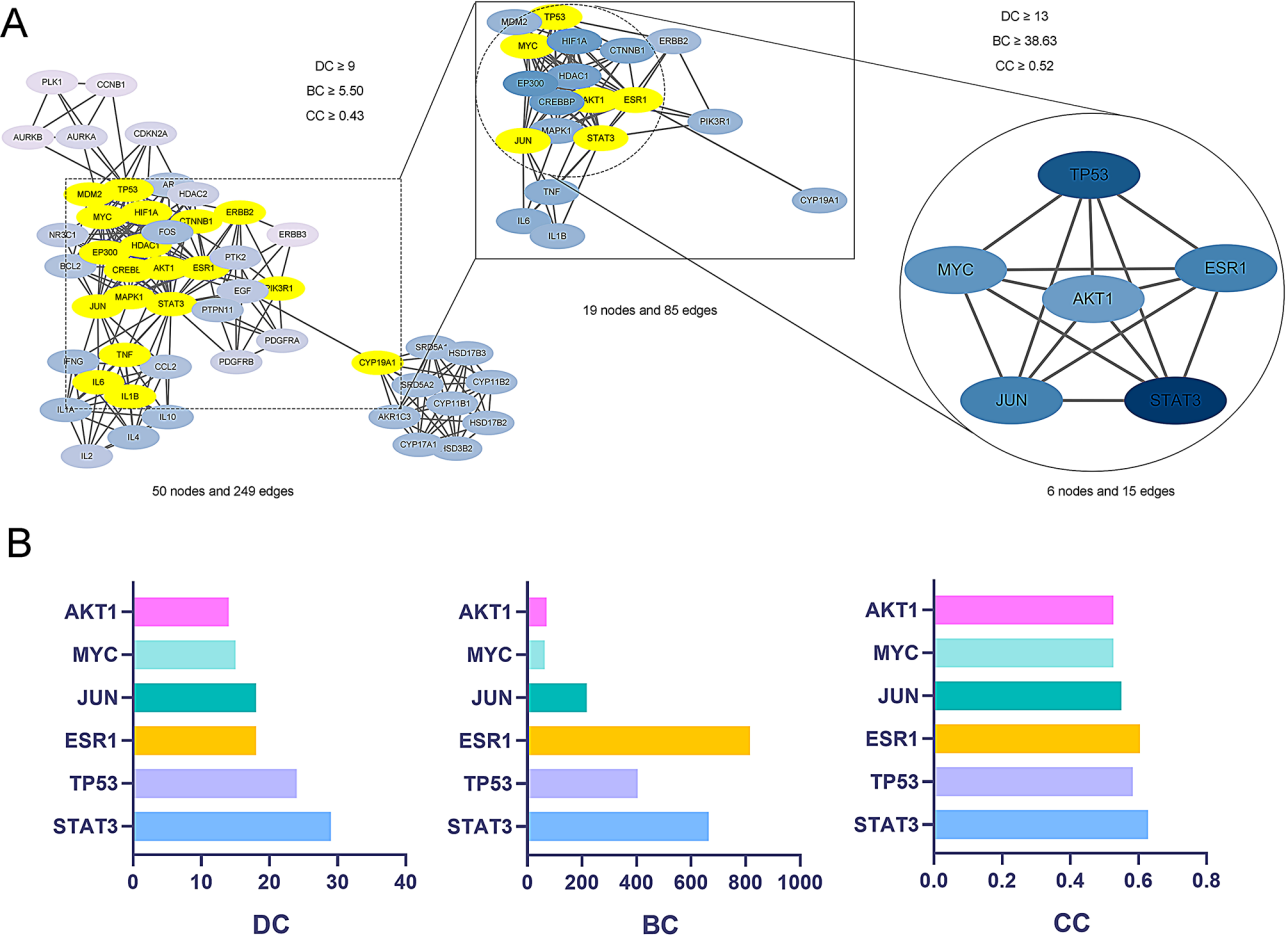
Screening of key targets of MDGLHD in CPP treatment. **A**. Screening of key targets using cytoNCA plug-ins of cytoscope software. **B**. The bar charts show degree centrality (DC), betweenness centrality (BC) and closeness centrality (CC) of 6 key targets

A PPI network of these 318 targets was obtained with STRING database, and a network of 316 nodes and 985 edges was obtained by hiding independent nodes (Fig S1). Visualization was performed with Cytoscope software, and 7 clusters with the scores greater than 3 were obtained, and labeled with 7 colors (Fig. 3A). Cluster 1 and cluster 2 had the highest scores (Fig. 3B), and the 50 genes involved in these two clusters were considered to be the core targets. The PPI network of these 50 genes was then obtained with STRING database (Fig. 3C). GO analysis showed that these targets were involved in “transcriptional regulation of gene expression”, “negative regulation of apoptosis”, and “positive regulation of cell proliferation” (Fig. 3D). KEGG enrichment analysis showed that these genes were mainly involved in “JAK-STAT signaling pathway” and “HIF-1 signaling pathway” (Fig. 3E and Table 2). Next, Cytoscope’s cytoNCA plug-in was used to calculate the BC, DC and CC of the 50 targets, screened twice by the median, and finally, a network of 6 nodes and 15 edges was obtained, which consited of signal transducer and activator of transcription 3 (STAT3), tumor protein p53 (TP53), estrogen receptor 1(ESR1), Jun proto-oncogene (JUN), MYC proto-oncogene (MYC) and AKT serine/threonine kinase 1 (AKT1) (Fig. 4A-B), and they were considered to be key targets for MDGLHD in CPP treatment.

**Table 2:** KEGG pathway enrichment results

Term	Count	%	PValue	Genes
hsa05200: Pathways in cancer	28	56	1.35E-20	HDAC2, HDAC1, PIK3R1, HIF1A, MYC, ERBB2, AKT1, MAPK1, EP300, PDGFRB, PDGFRA, JUN, CREBBP, CDKN2A, EGF, STAT3, FOS, ESR1, IL2, PTK2, IL4, AR, IL6, IFNG, BCL2, MDM2, CTNNB1, TP53
hsa05215: Prostate cancer	15	30	9.12E-17	PDGFRB, PDGFRA, CREBBP, SRD5A2, EGF, PIK3R1, AR, ERBB2, MDM2, BCL2, AKT1, EP300, MAPK1, CTNNB1, TP53
hsa04630: JAK-STAT signaling pathway	16	32	8.45E-15	IL10, PDGFRB, PDGFRA, CREBBP, EGF, STAT3, PTPN11, PIK3R1, IL2, IL4, IL6, IFNG, MYC, BCL2, AKT1, EP300
hsa04919: Thyroid hormone signaling pathway	13	26	1.94E-12	CREBBP, HDAC2, HDAC1, PIK3R1, HIF1A, ESR1, MYC, MDM2, AKT1, EP300, MAPK1, CTNNB1, TP53
hsa05205: Proteoglycans in cancer	15	30	3.72E-12	STAT3, PTPN11, PIK3R1, HIF1A, ESR1, TNF, PTK2, ERBB3, MYC, ERBB2, MDM2, AKT1, MAPK1, CTNNB1, TP53
hsa01522: Endocrine resistance	12	24	4.58E-12	JUN, CDKN2A, ERBB2, MDM2, BCL2, MAPK1, AKT1, FOS, PIK3R1, ESR1, TP53, PTK2
hsa05142: Chagas disease	12	24	7.16E-12	IL10, IL6, JUN, IFNG, IL1B, MAPK1, CCL2, AKT1, FOS, PIK3R1, TNF, IL2
hsa05418: Fluid shear stress and atherosclerosis	13	26	1.03E-11	JUN, PIK3R1, FOS, TNF, PTK2, IL1A, IFNG, IL1B, BCL2, CCL2, AKT1, CTNNB1, TP53
hsa05163: Human cytomegalovirus infection	15	30	1.32E-11	PDGFRA, CDKN2A, STAT3, PIK3R1, TNF, PTK2, IL6, MYC, IL1B, MDM2, CCL2, AKT1, MAPK1, CTNNB1, TP53
hsa01521: EGFR tyrosine kinase inhibitor resistance	11	22	1.40E-11	PDGFRB, PDGFRA, IL6, ERBB3, EGF, ERBB2, STAT3, BCL2, MAPK1, AKT1, PIK3R1
hsa04066: HIF-1 signaling pathway	12	24	1.50E-11	IL6, CREBBP, IFNG, EGF, ERBB2, STAT3, BCL2, EP300, MAPK1, AKT1, PIK3R1, HIF1A
hsa00140: Steroid hormone biosynthesis	10	20	4.52E-11	HSD3B2, SRD5A2, CYP11B2, SRD5A1, CYP11B1, HSD17B2, AKR1C3, HSD17B3, CYP19A1, CYP17A1
hsa05161: Hepatitis B	13	26	6.32E-11	CREBBP, JUN, STAT3, PIK3R1, FOS, TNF, IL6, MYC, BCL2, AKT1, EP300, MAPK1, TP53
hsa05321: Inflammatory bowel disease	10	20	7.03E-11	IL10, IL4, IL1A, IL6, JUN, IFNG, IL1B, STAT3, TNF, IL2
hsa05206: MicroRNAs in cancer	16	32	7.80E-11	PDGFRB, PDGFRA, CREBBP, HDAC2, CDKN2A, HDAC1, STAT3, PIK3R1, ERBB3, MYC, ERBB2, MDM2, BCL2, EP300, MAPK1, TP53

### Results of molecular Docking

**Fig. 5:**
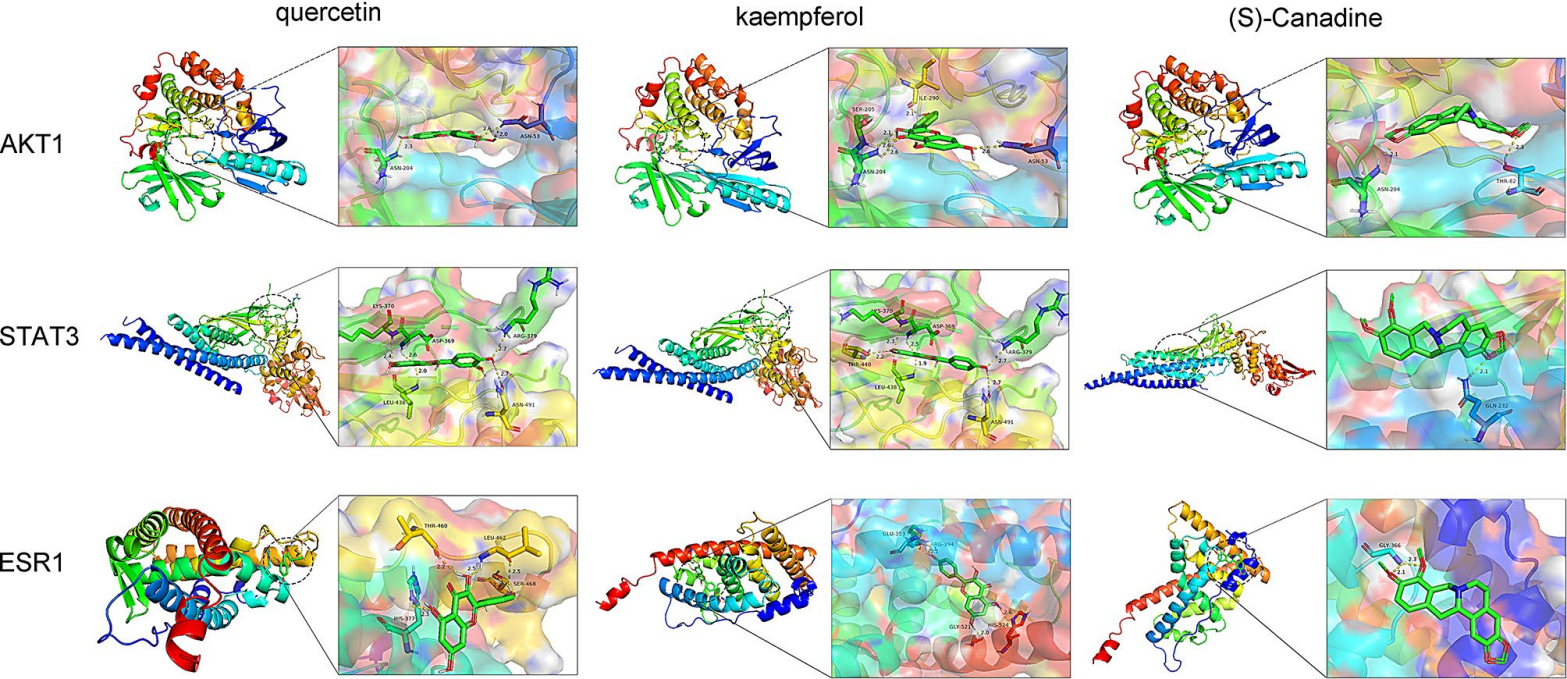
Molecular docking was applied for investigating the interactions between the bioactive components and the targets. **A**. Interactions between quercetin with AKT1, STAT3, and ESR1. **B**. Interactions between kaempferol with AKT1, STAT3 and ESR1. **C**. Interactions between (s)-canadine with AKT1, STAT3 and ESR1

We then explored the interactions between the top 3 compounds of MDGLHD [quercetin, kaempferol and (s)-canadine] and key targets (STAT3, TP53, ESR1, JUN, MYC and AKT1) through molecular docking. The results of molecular docking showed that the components of MDGLHD and the core targets had good binding activity with each other (Table 3). Quercetin formed three hydrogen bonds with ASN53 and ASN204 amino acid residues of AKT1; formed 5 hydrogen bonds with LYS370, ASP369, LEU438, ARG379 and ASN491 amino acid residues of STAT3; formed five hydrogen bonds with THR460, LEU462, SER468 and HIS377 amino acid residues of ESR1 (Fig. 5A). Kaempferol formed 5 hydrogen bonds with AKT1 amino acid residues ILE290, SER205, ASN204 and ASN53; form 6 hydrogen bonds with LYS370, ASP369, LEU438, ARG379, THR440 and ASN491 amino acid residues of STAT3; five hydrogen bonds were formed between it and GLU353, ARG394, GLY521 and HIS524 amino acid residues of ESR1 (Fig. 5B). (s)-canadine formed two hydrogen bonds with THR82 and ASN204 amino acid residues of AKT1; it formed a hydrogen bond with GLN232 amino acid residue of STAT3; formed two hydrogen bonds with GLY366 amino acid residue of ESR1 (Fig. 5C).

**Table 3:**
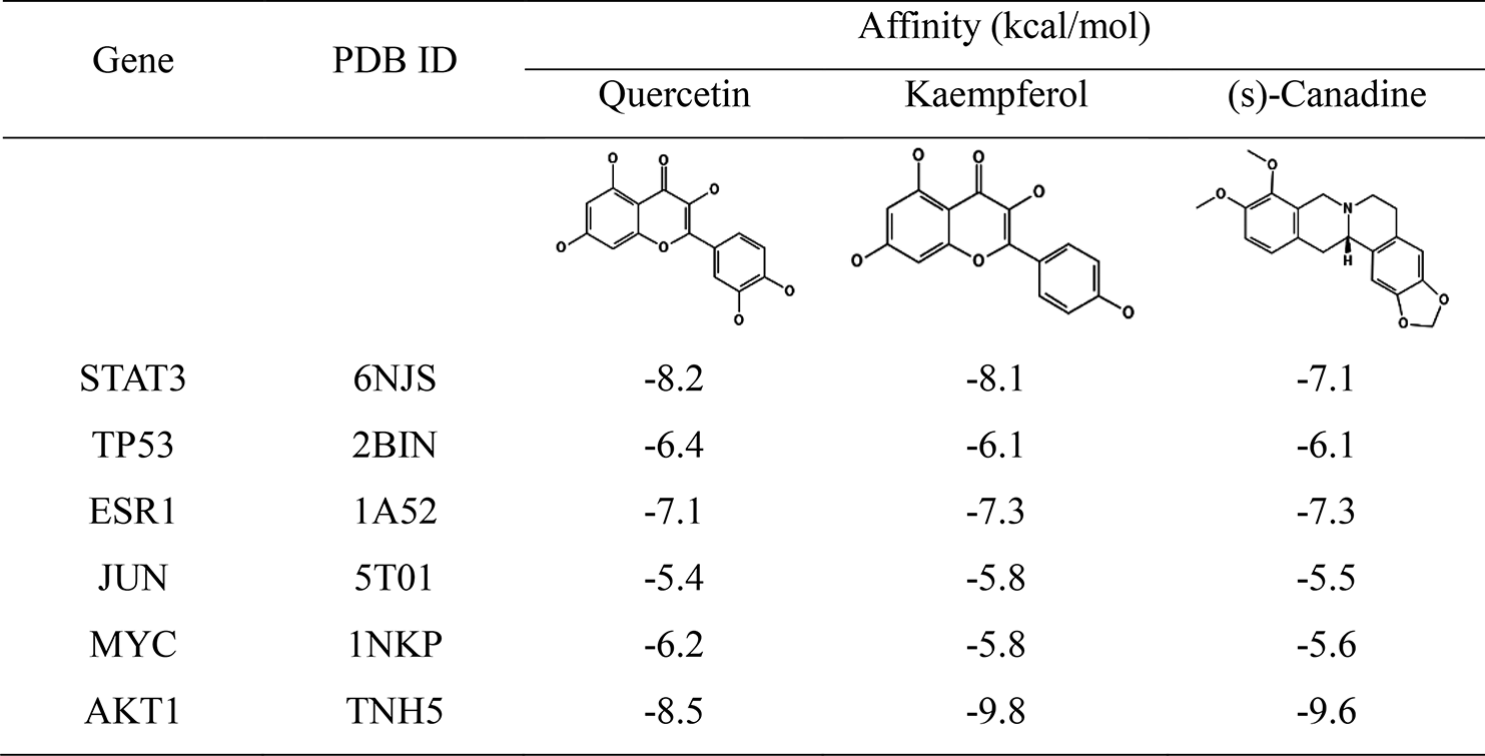
The affinity of ingredients with key targets

### MDGLHD inhibits the expression of genes related to GnRH secretion and activity of neuroendocrine signals

**Fig. 6:**
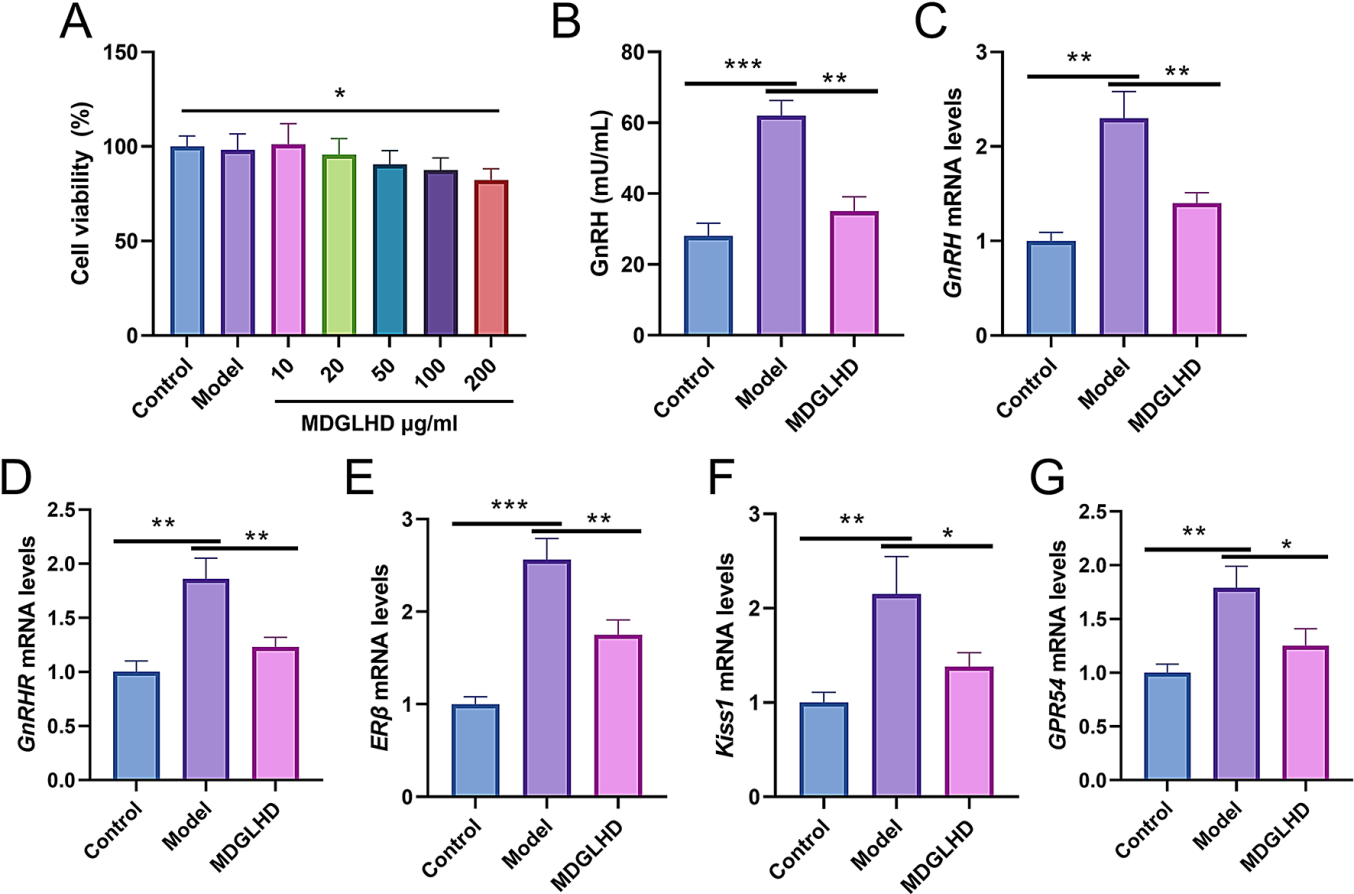
MDGLHD extract inhibits the expression of genes related to estrogen secretion and neuroendocrine signals. **A**. Effect of MDGLHD extract on the viability of GT1-7 cells was evaluated by CCK-8 assay. **B**. The level of GnRH in the supernatant of the cells of each group was detected by ELISA. **C**-**G**. The mRNA expression levels of GnRH (**C**), GnRHR (**D**), ERβ (**E**), Kiss1 (**F**) and GPR54 (**G**) in GT1-7 cells of each group were detected by qRT-PCR. *P < 0.05, **P < 0.01, ***P < 0.001

In order to further explore the mechanism of action of MDGLHD on CPP, we established an in vitro model of GT1-7 cells treated with E2, and then the cells were treated with MDGLHD extract to explore its effect on GnRH secretion. CCK-8 assay was used to detect the toxicity of MDGLHD on GT1-7 cells. The results showed that when the concentration of MDGLHD extract reached 200 µg/ml, the viability of GT1-7 cells was decreased significantly compared with the control group; however, the viability of cells in the model group treated with 100 µg/ml extact was not significantly different from that in the control group (Fig. 6A). So in the following experiments, 100 µg/ml MDGLHD extract was used. ELISA showed that the level of GnRH in the model group was increased significantly compared with the control group; and GnRH level in MDGLHD treatment group was significantly lower than that in the model group (*P* < 0.05) (Fig. 6B). qRT-PCR showed that GnRH, gonadotropin-releasing hormone receptor (GnRHR), estrogen receptor β (ERβ), kiss-1 metastasis-suppressor (KISS1), and G-protein coupled receptor 54 (GPR54) mRNA expression levels in GT1-7 cells treated with MDGLHD were significantly lower than those in the model group (Fig. 6C-G). These results suggest that MDGLHD exerts a therapeutic effect on CPP by inhibiting the expression of GnRH and neuroendocrine signaling receptor genes.

## Discussion

DGLHD is a TCM prescription with “heat-clearing” properties, and in China, DGLHD is widely used in treating diseases related to hormone disorders, such as hyperthyroidism and perimenopausal syndrome [27, 36]. MDGLHD is an improved version of DGLHD, which addition of grilled turtle plate, rhizoma anemarrhenae, and cortex moutan. Network pharmacology is an emerging discipline based on the combination of bioinformatics, network biology and pharmacology [37]. This study explores the potential molecular mechanism of MDGHLD therapy for CPP based on network pharmacology.

According to the drug-compound-target network, quercetin, kaempferol, and (s)-canadine were screened, and considered to be the most important bioactive ingredient in MDGLHD. Quercetin and kaempferol are flavonols that are widely distributed in fruits and vegetables. They have a variety of pharmacologic effects, including cardioprotective, neuroprotective, immunoregulatory, anti-tumor, antioxidant, antibacterial and anti-inflammatory effects, etc., which play an important role in age-related diseases [38, 39]. Notably, quercetin and kaempferol have properties of phytoestrogens, and the molecular structure of phytoestrogens is similar to that of mammalian estrogens. Phytoestrogens can play physiological roles in simulating, interfering and bidirectional regulation of endocrine levels [40–43]. Phytoestrogen has a wide range of effects on hormone-related diseases, especially on the prevention of breast cancer, mammary hyperplasia, uterine fibroids, prostate cancer, menopausal syndrome, cardiovascular diseases and osteoporosis [44–47]. (s)-Canadine is an alkaloid with pharmacologic effects such as anti-oxidation [48], anti-platelet aggregation [49], anti-Alzheimer’s disease [50, 51], and anti-muscle atrophy [52]. In addition, Canadine can promote the activation of Akt signaling [45], and notably PI3K/Akt signaling pathway is one of the important pathways to regulate GnRH expression and secretion [53, 54]. The data from this study preliminarily demonstrate the main components of MDGLHD in the treatment of CPP and suggest the direction of future research.

In this work, we obtained 318 potential targets of MDGLHD in CPP treatment. Subsequent analysis suggested that MDGLHD could probably treat CPP through two signaling pathways, JAK-STAT signaling pathway and HIF-1 signaling pathway. A study of goat pituitary DEGs at different developmental stages showed that JAK-STAT signaling pathway is closely related to HPG and early puberty [55]. Female GnRH neuron-specific JAK2 conditioned knockout mice (JAK2 G-/-) showed decreased GnRH expression and neuronal activity, and female JAK2 G-/- mice showed delayed puberty and decreased fertility. These studies imply that JAK-STAT signaling pathway plays a crucial role in the regulation of GnRH neurons during puberty [56]. In addition, modulating the JAK-STAT signaling pathway may prevent CPP associated with obesity [57]. HIF-1 signaling pathway is involved in regulating FSH-induced follicular angiogenesis, affecting follicle survival, ovulation, and oocyte development [58]. In addition, HIF-1α is an important regulator of ovarian luteal development in mammals, and is related to the secretion of progesterone and ovarian prostaglandin content [59]. The pharmacological effects of MDGLHD on JAK-STAT and HIF-1 signaling pathway remain to be further confirmed by in vitro and in vivo experiments.

Subsequently, we constructed a PPI network and six core targets were identified through topological analysis, namely STAT3, TP53, ESR1, JUN, MYC and AKT1. STAT3 is a transcription factor that mediates extracellular signaling, such as cytokines and growth factors, through interactions with cell surface polypeptide receptors [60]. Activated STAT3 can bind to sequence-specific DNA elements to transcribe target genes, and regulate the balance of sex hormones [61]. AKT1 is a serine/threonine protein kinase. AKT can regulate the synthesis and release of pituitary gonadotropin by luteinizing hormone releasing hormone (LHRH) receptors, as well as the proliferation and apoptosis of pituitary cells [62]. Kiss-1/GPR54 can be activated by the STAT3/p53 and PI3K/Akt/mTOR signaling pathways, to promote the release of GnRH in the hypothalamus [54, 63]. ESR1, encoding estrogen receptor (ER), is closely related to the development and CPP [64, 65]. Previous studies have confirmed that TP53, ESR1, JUN and AKT1 are key genes in “nourishing yin and purging fire” (NYPF) therapy for CPP [57, 66]. Overexpression of p53 in the hypothalamus may accelerate HPG activation through c-MYC [67]. These findings all prove that STAT3, TP53, ESR1, JUN, MYC and AKT1 play an important role in CPP, which are probably targeted by MDGLHD.

The pathogenesis of CPP is related to the early initiation of HPG, the key factor of which is pulsatile secretion of GnRH. It is known that the combination of GPR54 protein and kisspeptin encoded by KISS1 gene stimulates hypothalamic neurons to release GnRH, causing the pituitary to secrete gonadotrophins (LH and FSH) and sex steroids, which then act on the gonads to produce gametes [68]. KISS1 and its receptor GPR54 therefore play an integral role in the initiation of puberty and reproduction [69, 70]. ERβ can directly participate in estrogen regulation by regulating neuronal activity, gene expression and pulsatile secretion of GnRH [71]. To investigate the effect of MDGLHD on HPG, in the present work we treated GT1-7 cells with E2 to construct an in vitro model. The results showed that MDGLHD could inhibit the secretion of GnRH and the expression of GnRH, GnRHR, ERβ, KISS1, and GPR54 mRNA. As mentioned above, STAT3/p53 and PI3K/Akt/mTOR signaling activate KISS1/GPR54, to modulate the release of GnRH [54, 63], considering the results of molecular docking suggested that the main bioactive components of MDGLHD had good binding affinities with STAT3, TP53 and AKT1, MDGLHD probably regulates GnRH synthesis and secretion via repressing STAT3/p53 and PI3K/Akt/mTOR signaling and reducing the expression levels of KISS1 and GPR54. In the future, the properties of these bioactive components as the natural inhibitor of these pathways require further verification with computational biology and molecular biology approaches.

## Conclusion

The main active components of MDGLHD in CPP treatment were quercetin, kaempferol, and (S)-Canadine, and the main targets were STAT3, TP53, ESR1, JUN, MYC and AKT1. MDGLHD extract can inhibit GnRH secretion and neuroendocrine signaling. The mechanism of MDGLHD in CPP treatment was preliminarily revealed, which provides a certain theoretical basis for the clinical application of MDGLHD. However, animal experiments and clinical trails are still needed to further verify the safety, efficacy, and mechanism of MDLHD in CPP treatment.

## Electronic supplementary material

Below is the link to the electronic supplementary material.

Supplementary Material 1: Supplementary Fig.1. The PPI network of MDGLHD targets in CPP treatment was obtained with String database. Supplementary Material 1: Supplementary Fig.1. The PPI network of MDGLHD targets in CPP treatment was obtained with String database.

Supplementary Material 2: Supplementary Table 1. Herbs and Ingredients of Modified Danggui Liuhuang Decoctions.

## Data Availability

The data used to support the findings of this study are available from the corresponding author upon request.
