# Supplementary figures and images for: Exploration of the molecular mechanism of modified Danggui Liuhuang Decoction in treating central precocious puberty and its effects on hypothalamic-pituitary-gonadal axis hormones
Source: Hereditas. 2025 Apr 8;162:56. doi: 10.1186/s41065-025-00420-9 (PMC11980125; doi:10.1186/s41065-025-00420-9)

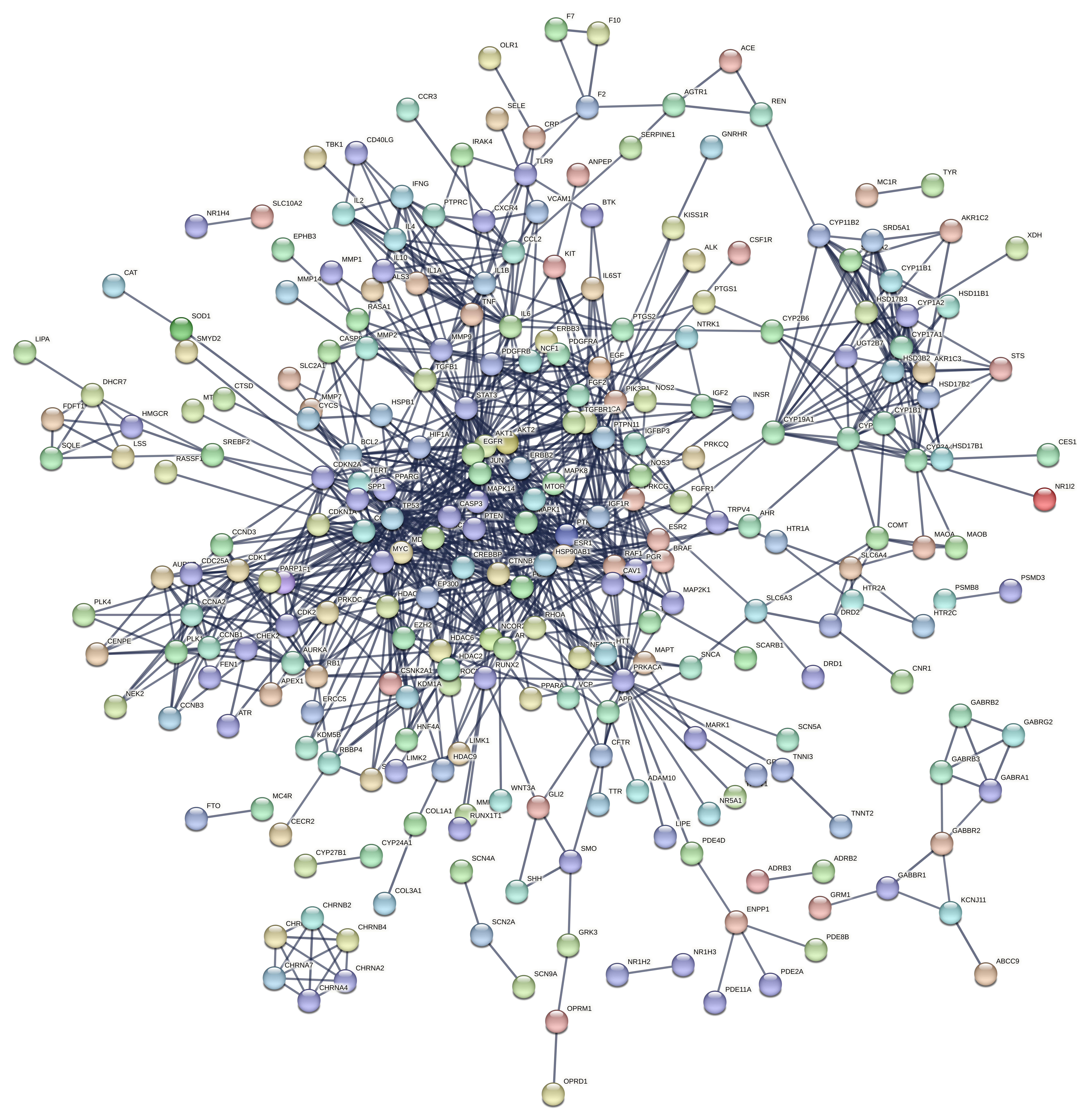

Supplement: Supplementary file 1 — Supplementary Material 1: Supplementary Fig.1. The PPI network of MDGLHD targets in CPP treatment was obtained with String database. [file 41065_2025_420_MOESM1_ESM.jpg]
